# Case Report: Metabolic alterations and cholesterol esterification in a low-grade diffuse astrocytoma patient who progressed to glioblastoma at recurrence

**DOI:** 10.3389/fonc.2025.1651974

**Published:** 2025-09-19

**Authors:** Omkar B. Ijare, David S. Baskin, Suzanne Z. Powell, Kumar Pichumani

**Affiliations:** ^1^ Kenneth R. Peak Brain and Pituitary Tumor Treatment Center, Department of Neurosurgery, Houston Methodist Hospital, Houston, TX, United States; ^2^ Houston Methodist Academic Institute, Houston, TX, United States; ^3^ Houston Methodist Research Institute, Houston, TX, United States; ^4^ Houston Methodist Neurological Institute, Houston, TX, United States; ^5^ Weill Cornell Medical College, New York, NY, United States; ^6^ Texas A&M University College of Medicine, Houston, TX, United States; ^7^ Department of Pathology and Genomic Medicine, Houston Methodist Hospital, Houston, TX, United States

**Keywords:** alanine, cholesterol, cholesteryl ester, diffuse astrocytoma, glioblastoma, magnetic resonance spectroscopy, TERT promoter mutation

## Abstract

**Background:**

Metabolic alterations during transformation of low-grade gliomas (LGGs) into high-grade glioblastomas (GBMs) remain incompletely understood. Particularly, IDH wildtype (IDHwt) diffuse astrocytomas harboring TERT promoter (TERTp) mutations, classified as molecular GBM under the 2021 WHO classification, may exhibit distinct metabolic and epigenetic features compared to histological WHO Grade 4 GBMs. Here, we conducted a detailed metabolomic comparison of tumor specimens from a patient initially diagnosed with WHO Grade 2 IDHwt diffuse astrocytoma carrying TERTp mutation, who subsequently progressed to a histologically confirmed WHO Grade 4 GBM upon recurrence.

**Case presentation:**

A 66-year-old female patient underwent surgical resection of a WHO Grade 2 diffuse astrocytoma in April 2018. Molecular testing revealed IDH1-wildtype status and TERTp mutation, classifying the tumor as a molecular GBM. Following ~3.5 years of clinical stability, magnetic resonance imaging detected tumor recurrence. The patient underwent a second craniotomy in February 2022, with histopathology confirming progression to WHO Grade 4 GBM. Using untargeted proton nuclear magnetic resonance (^1^H NMR) spectroscopy, we analyzed aqueous-methanol and chloroform phases from methanol-chloroform-water extraction of tumor tissue from both time points. Compared to the primary tumor, the aqueous-methanol phase of the recurrent WHO Grade 4 GBM specimen showed decreased levels of neuronal and glial markers including N-acetylaspartate, myo-inositol, and scyllo-inositol. Elevated metabolites included phosphocholine, phosphoethanolamine, glycine, taurine, hypotaurine, branched-chain amino acids (leucine, isoleucine, valine), and notably alanine, which increased approximately 6-fold. Alanine likely serves as an alternative carbon source supporting tumor proliferation and aggressiveness. The chloroform phase showed the presence of cholesterol in both tumors; however, cholesteryl ester (CE) was detected only in the recurrent tumor. The CE-to-cholesterol ratio of 0.44 in the recurrent tumor suggests significant cholesterol esterification during malignant progression.

**Conclusion:**

Our findings identify alanine accumulation and increased cholesterol esterification as key metabolic features accompanying malignant transformation from molecular GBM to histological WHO grade-4 GBM. These metabolic changes may serve as biomarkers of tumor progression and recurrence. Importantly, alanine detection via magnetic resonance spectroscopy offers promising potential for non-invasive glioma diagnostics. Furthermore, targeting cholesterol esterification pathways using Acyl-CoA:cholesterol acyltransferase inhibitors could provide a novel therapeutic approach, especially for low-grade astrocytomas with high risk of malignant progression and recurrence.

## Introduction

Low-grade gliomas (LGGs) are a heterogeneous group of primary brain tumors classified by the World Health Organization (WHO) into Grades 1 through 3. Common subtypes include MYB- or MYBL1-altered diffuse astrocytomas (WHO Grade 1), IDH-mutant astrocytomas (WHO Grade 2 or 3), and oligodendrogliomas with IDH mutations and 1p/19q-codeletion (WHO Grade 2 or 3) (1). According to the 2021 WHO classification, certain IDH-wildtype (IDHwt) diffuse astrocytomas that harbor molecular alterations such as high-level EGFR amplification with chromosome 7 gain and 10 loss (7+/10−), or TERT promoter (TERTp) mutations, are now categorized as “diffuse astrocytoma, IDH-wildtype, with molecular features of glioblastoma (GBM)”—commonly referred to as molecular GBM (1–3).

However, it is important to emphasize that molecular GBM is not biologically or clinically identical to histological WHO Grade 4 GBM. In particular, the metabolic and epigenetic characteristics of these tumors can differ significantly. While both may share features of aggressive growth and poor prognosis, their distinct biology underscores the need for dedicated molecular and metabolic investigations. This distinction is critical for developing effective, subtype-specific therapies.

LGGs carry a risk of recurrence after surgical resection, influenced by molecular features such as IDH mutation status, 1p/19q-codeletion, and TERTp mutations (4, 5). GBM, a histologically defined WHO Grade 4 malignant central nervous system (CNS) tumor, represents 14.2% of all CNS tumors in the United States, with an annual incidence of 3.27 per 100,000 and a median survival of only ~8 months (6). Current standard-of-care therapies—surgery, radiation, and chemotherapy—offer limited benefit (median survival <16 months) and are associated with poor quality of life (7), emphasizing the need for novel therapeutic approaches for both LGGs and GBMs.

Gliomas are known to reprogram key metabolic pathways—including glycolysis, mitochondrial oxidative phosphorylation, glutaminolysis, and fatty acid and cholesterol metabolism—to fuel tumor growth and progression (8).

In this study, we report metabolomic profiling of tumor specimens from a patient initially diagnosed with diffuse astrocytoma (WHO Grade 2) harboring a TERTp mutation, who experienced tumor recurrence and histological progression to WHO Grade 4 GBM approximately 3.5 years after the first surgery. We identified specific metabolic alterations—particularly a 6-fold increase in alanine levels and dysregulation of cholesterol metabolism—between the initial and recurrent tumors.

To our knowledge, this is the first study to longitudinally compare tumor metabolomic profiles in a patient with molecular GBM (WHO Grade 2 with TERTp mutation) that later progressed to histological WHO Grade 4 GBM. This case highlights the critical need to metabolically characterize molecular GBMs—such as WHO Grade 2 diffuse astrocytomas with TERTp mutations—independently from histological WHO Grade 4 GBMs, as their distinct metabolic vulnerabilities may offer unique therapeutic opportunities and guide more precise treatment strategies.

## Case description

**Figure 1:**
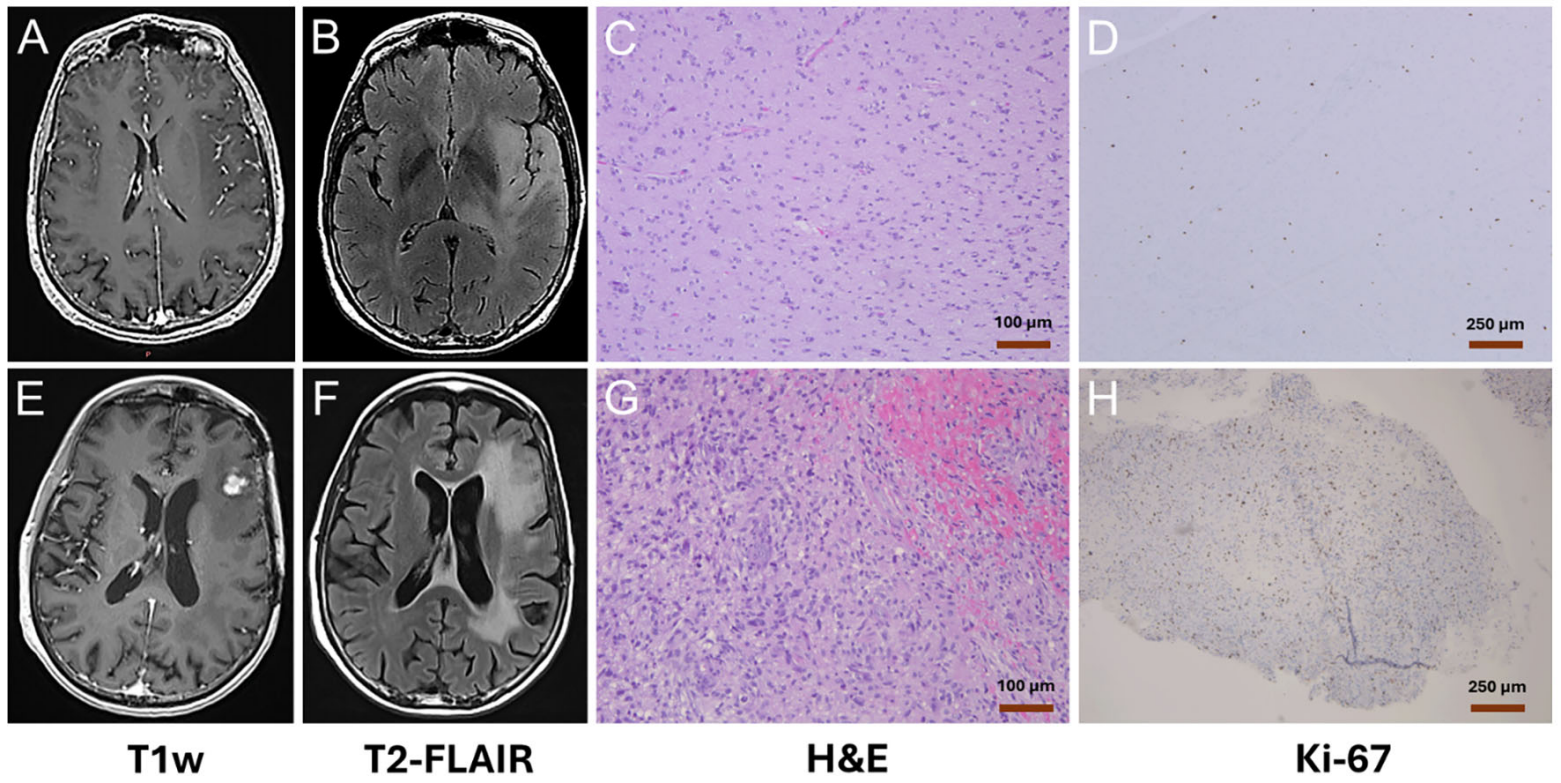
MRI and immunohistochemical characterization of the primary and recurrent gliomas. **(A)** Post-contrast axial T1-weighted (T1w) and **(B)** T2-FLAIR MRI of the primary tumor (WHO Grade 2 diffuse astrocytoma) demonstrate a non-enhancing, diffusely infiltrative lesion in the upper left temporal lobe. T2-FLAIR reveals extensive hyperintensity with associated mass effect on the left lateral ventricle and mild midline shift. **(C)** H&E and **(D)** Ki-67 immunohistochemical staining of the resected tumor show diffuse astrocytic infiltration and a low proliferation index (Ki-67: ~5%), consistent with WHO Grade 2 diffuse astrocytoma. **(E)** Post-contrast T1w and **(F)** T2-FLAIR MRI of the recurrent tumor ~3.5 years later reveal a new contrast-enhancing lesion in the left inferior frontal gyrus and expanded FLAIR hyperintensity extending to the left temporal lobe and periventricular region. **(G)** H&E and **(H)** Ki-67 staining of the recurrent tumor demonstrate increased cellular density and elevated proliferative activity (Ki-67: 10–13%), confirming progression to high-grade glioma (WHO Grade 4 glioblastoma). (Magnifications: H&E, 10×; Ki-67, 4×).

A 66-year-old woman presented with an infiltrating brain neoplasm and underwent tumor resection in April 2018. Preoperative MRI showed no contrast enhancement on T1-weighted imaging (**Figure 1A**), but T2-FLAIR revealed a large hyperintense region in the left temporal lobe (**Figure 1B**), suggestive of non-enhancing infiltrative glioma. Histopathological examination identified a diffuse astrocytoma with a proliferation index (Ki-67) of 5% (**Figures 1C, D**), and no evidence of necrosis or microvascular proliferation, consistent with WHO Grade 2 (2016 classification).

Molecular testing showed IDH1 wildtype status, TERT promoter (TERTp) mutation, and unmethylated MGMT promoter status, indicating a likely aggressive clinical course. Although these molecular features would now classify the tumor as a molecular glioblastoma per the 2021 WHO criteria, at the time of diagnosis, the cIMPACT-NOW update 3 had not yet been published (released in November 2018), and the tumor was classified as a diffuse astrocytoma, IDH-wildtype, TERTp-mutant.

Following surgery, the patient received standard chemoradiation with temozolomide (TMZ, 75 mg/m²) and 60 Gy radiation in 30 fractions with 2 Gy per fraction (May–July 2018), followed by six cycles of adjuvant TMZ (150 mg/m², August 2018–January 2019). She tolerated the treatment well and remained clinically stable and asymptomatic. Serial MRI scans from July 2018 to September 2021 showed no evidence of tumor progression.

In January 2022 (~3.5 years later), MRI revealed a new 1.6 cm contrast-enhancing lesion in the left inferior frontal gyrus (**Figure 1E**), with associated T2-FLAIR hyperintensity (**Figure 1F**). *In vivo* proton magnetic resonance spectroscopy (^1^H MRS) showed elevated choline and decreased NAA, while ^18^F-FDG PET demonstrated increased uptake of ^18^F-FDG near the lesion (**Supplementary Figure S1**), showing upregulated metabolic activity, consistent with high-grade glioma. A second craniotomy in February 2022 confirmed a high-grade glial neoplasm with Ki-67 of 10–13%, diagnostic of WHO Grade 4 glioblastoma.

After a second round of chemoradiation, the patient remained clinically stable until November 2022, when she experienced seizures, a fall, skull fracture, and aphasia. In March 2023, MRI identified a second recurrence in the left frontal cortex (1.4 cm lesion), which was treated with single-dose Gamma Knife radiosurgery (18 Gy). The patient tolerated the procedure without complications.

In June 2023, she developed bilateral lower limb weakness and required emergency treatment. She subsequently transitioned to home-based palliative care and passed away in October 2023.

## *Ex vivo* ^1^H magnetic resonance spectroscopy

**Figure 2:**
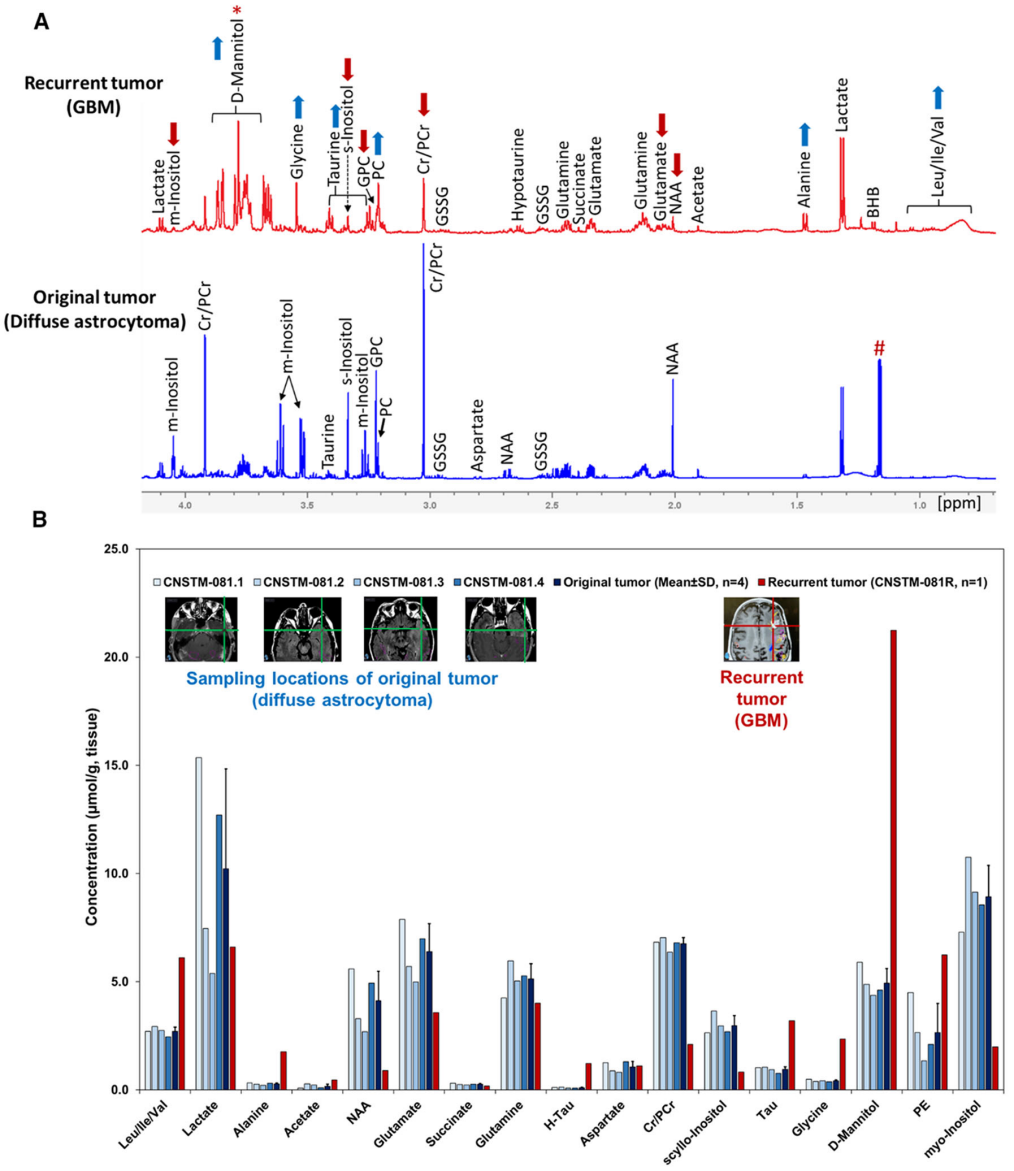
Comparative ¹H NMR metabolite profiles of aqueous extracts from primary and recurrent tumor specimens. **(A)** Representative ¹H NMR spectra of aqueous-phase extracts (methanol–chloroform-water extraction) from tumor tissues collected during the first surgery (April 2018; WHO Grade 2 diffuse astrocytoma with TERTp mutation (molecular GBM); bottom spectrum, blue) and second surgery following recurrence (February 2022; WHO Grade 4 GBM; top spectrum, red). The spectra reveal distinct metabolic alterations associated with tumor progression. **(B)** Bar graphs showing the metabolite concentrations (µmol/g, wet tissue) in tumor specimens collected from four different spatial locations of the original tumor (CNSTM-081.1 to CNSTM-081.4), the mean ± SD of metabolite concentrations (µmol/g wet tissue) in the primary tumor (*n* = 4) and the recurrent tumor (*n* = 1), showing the relative levels of metabolites in WHO Grade 2 molecular GBM and WHO Grade 4 GBM. Insets display axial T1-weighted MR images indicating tumor sampling locations. Characteristic metabolic features of high-grade gliomas—such as decreased levels of N-acetylaspartate (NAA), glycerophosphocholine (GPC), myo-inositol (mI), scyllo-inositol (sI), and elevated levels of phosphocholine (PC) and glycine—were observed in the recurrent tumor, consistent with progression from a WHO Grade 2 molecular GBM to histologically confirmed GBM. Additionally, Leu/Ile/Val, alanine, hypotaurine (H-Tau) and taurine (Tau) were elevated in the recurrent tumor. (*) D-Mannitol, an exogenous osmotic agent administered intraoperatively to reduce intracranial pressure, was detected in both tumor samples. Notably, its concentration was substantially higher in the recurrent tumor (21.25 µmol/g) than in the primary tumor (4.94 µmol/g), suggestive of increased blood-brain or blood-tumor barrier permeability in the high-grade recurrent tumor.

We collected tumor specimens from the patient during both the initial (primary) and second (recurrent) surgeries and performed metabolomic analyses using *ex vivo* high resolution proton nuclear magnetic resonance (^1^H NMR) spectroscopy. **Figure 2** presents the ^1^H NMR spectral profiles and quantified aqueous-phase metabolites (methanol-extracted) from the primary tumor—classified as WHO Grade 2 diffuse astrocytoma with TERT promoter (TERTp) mutation (molecular GBM)—and the recurrent tumor, diagnosed as WHO Grade 4 glioblastoma (GBM).

Compared to the primary tumor, the recurrent tumor exhibited markedly reduced levels of N-acetylaspartate (NAA), glycerophosphocholine (GPC), myo-inositol (mI), scyllo-inositol (sI), creatine (Cr), and glutamate. In contrast, levels of branched-chain amino acids (leucine/isoleucine/valine), alanine, phosphocholine (PC), glycine, taurine, hypotaurine (H-Tau), and phosphoethanolamine (PE) were elevated in the recurrent tumor specimen. Notably, decreased NAA, mI, and sI, alongside elevated PC and glycine, are well-established metabolic hallmarks of high-grade gliomas (9–11). Elevated valine, alanine, taurine, and H-Tau levels have also been reported in high-grade gliomas (12–14). Amino acid metabolism is increasingly recognized as a critical contributor to glioma progression (13, 14). In our preliminary studies, using (3-^13^C)alanine as a metabolic tracer, we observed incorporation of labeled carbon into TCA cycle intermediates, including acetyl-CoA, citrate, and malate, suggesting that alanine contributes to energy metabolism and supports biosynthesis of glutamate and aspartate—amino acids essential for tumor cell proliferation (15).

These metabolomic changes strongly support progression from WHO Grade 2 diffuse astrocytoma to Grade 4 GBM over ~3.5 years and align with the histopathological features observed in the recurrent tumor (**Figures 1G, H**). Although the cIMPACT-NOW update 3 (published in November 2018) recommended that diffuse astrocytic gliomas harboring IDH-wildtype and TERTp mutation be considered molecular GBMs (WHO Grade 4), the metabolic profile of the primary tumor resembled that of normal-appearing brain tissue, distinct from that of a typical GBM (16).

**Figure 3:**
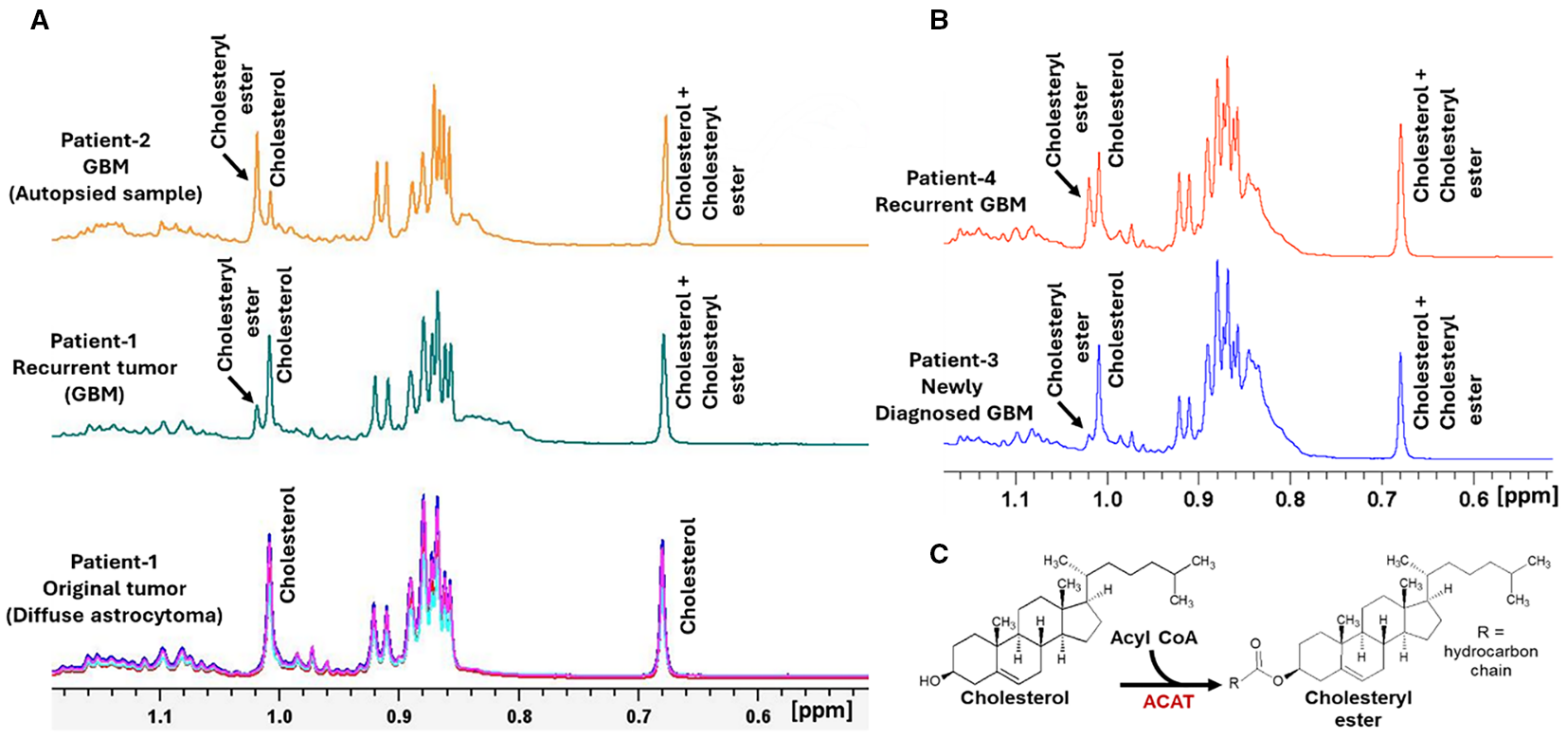
Increased cholesterol esterification in primary and recurrent gliomas. **(A)** Comparison of ¹H NMR spectra (0.52 – 1.18 ppm region showing H-18 and H-21 proton peaks) from the chloroform-phase extracts of tumor tissue specimens collected during the initial surgery (April 2018; WHO Grade 2 diffuse astrocytoma; bottom spectral overlay, shown in red, turquoise, pink and blue, n = 4), the second surgery for the recurrent tumor (February 2022; WHO Grade 4 GBM; middle spectrum, shown in green), and from an autopsied tissue specimen from a deceased end-stage GBM patient (Patient-2; top spectrum, shown in orange). While cholesterol signals were present in both primary and recurrent tumors, cholesteryl ester **(CE)** peaks were detected exclusively in the recurrent and autopsy samples. Notably, the CE signal was markedly elevated in the autopsied GBM tissue, suggesting progressive accumulation of cholesteryl esters (CEs) during disease advancement. **(B)** Representative ¹H NMR spectra from chloroform-phase extracts of tumor tissues from a newly diagnosed GBM patient (Patient-3) and a recurrent GBM patient (2 years post-surgery, Patient-4) demonstrate increased CE levels in the recurrent tumor (Patient-4), supporting enhanced cholesterol esterification during recurrence and progression. **(C)** Schematic illustration of the enzymatic conversion of cholesterol to cholesteryl ester (CE) catalyzed by acyl-CoA:cholesterol acyltransferase (ACAT), a key metabolic step implicated in glioma progression.

We further analyzed the chloroform-phase for lipid extracts from both tumor specimens. Cholesterol was present in both the primary and recurrent tumors (**Figure 3A**, bottom and middle spectra), but cholesteryl ester (CE) was detected exclusively in the recurrent GBM (middle spectrum, shown in green). The CE-to-cholesterol ratio in the recurrent tumor was 0.44, indicating that approximately 30% of the total cholesterol pool was esterified. These findings suggest the activation of *de novo* cholesterol esterification during malignant progression from molecular GBM to histological GBM.

To explore this further, we analyzed a postmortem GBM tissue sample from a second patient (Patient-2) with end-stage recurrent GBM. The ^1^H NMR spectrum of the chloroform extract (**Figure 3A**, top spectrum, shown in orange) revealed even higher CE levels than in Patient-1, with ~70% of the cholesterol pool esterified. This supports the notion that the cholesterol esterification pathway becomes increasingly active during GBM progression and advanced disease stages.

Finally, to determine whether CE accumulation is a generalizable feature of recurrence, we compared ^1^H NMR spectra of tumor lipid extracts from a newly diagnosed GBM patient (Patient-3) and a recurrent GBM patient (Patient-4; 2 years post-surgery; **Figure 3B**). CE levels were markedly elevated in the recurrent tumor, suggesting that cholesterol esterification is enhanced during recurrence and may be a metabolic marker of GBM progression.

## Discussion and conclusions

Although the 2021 WHO classification of CNS tumors recommends that IDHwt diffuse astrocytomas harboring TERTp mutation be classified as molecular glioblastomas (GBMs) (1), the metabolic profile of the original tumor in our patient did not align with that of a primary GBM (**Figure 2A**, bottom spectrum). Instead, the ¹H NMR spectrum of the primary tumor (a diffuse astrocytoma with TERTp mutation) resembled that of normal-appearing brain tissue, consistent with the lack of contrast enhancement on post-contrast T1-weighted MRI (**Figure 1A**). However, after approximately 3.5 years, the recurrent tumor exhibited a distinctly altered metabolic profile that matched the metabolic features of WHO Grade 4 GBM (**Figure 2A**, top spectrum), including elevated levels of phosphocholine (PC) and glycine, and reduced levels of N-acetylaspartate (NAA), scyllo-inositol (sI), and myo-inositol (mI). This metabolic shift was mirrored in the imaging, with new contrast enhancement observed on MRI (**Figure 1E**), consistent with high-grade transformation.

These findings suggest that ¹H NMR-based metabolomic profiling of tumor specimens can offer valuable insights into the biological grade of gliomas, including molecular subtypes and progression. Such “metabolic fingerprinting” or “metabotyping” may complement existing histopathological and molecular diagnostic tools and improve the differential diagnosis and prognostication of gliomas.

Amino acid metabolism is increasingly recognized as a key component of tumor biology, functioning not only as a source of biosynthetic precursors and energy substrates but also as modulators of signaling pathways (12, 13). In normal brain physiology, glycine supports multiple functions, including inhibitory neurotransmission, cytoprotection, protein synthesis, and one-carbon metabolism. Elevated glycine levels are consistently associated with high-grade gliomas, and glycine has been proposed as a non-invasive imaging biomarker of glioma malignancy (17, 18). In our study, glycine levels were 5-fold higher in the recurrent tumor than in the original lesion, supporting its high-grade status and aggressive potential.

Taurine and hypotaurine were also significantly elevated (3-fold and 12-fold, respectively) in the recurrent tumor. While taurine is considered neuroprotective and is often used to mitigate the side effects of chemotherapy, recent studies have shown that taurine may promote energy metabolism and tumor progression in leukemia (19). The biological significance of hypotaurine and taurine in glioma metabolism remains poorly understood and warrants further investigation.

Of particular interest, alanine—long overlooked as a passive byproduct—has emerged as a key bioenergetic substrate in cancer. In pancreatic ductal adenocarcinoma (PDAC), alanine released by stromal cells supports energy metabolism and amino acid biosynthesis in tumor cells (20). Our own prior work demonstrated that (3-¹³C)alanine serves as a nutrient in patient-derived GBM cells, contributing to tricarboxylic acid (TCA) cycle intermediates and the synthesis of nonessential amino acids such as glutamate and aspartate (15). In the present study, a 6-fold increase in alanine was observed in the recurrent tumor, suggesting that alanine may support metabolic plasticity and proliferation in high-grade gliomas. Notably, alanine has recently been proposed as both a diagnostic marker for IDHwt status and a prognostic indicator in GBM (14).

In addition to altered amino acid metabolism, we observed marked dysregulation of cholesterol metabolism in the recurrent tumor. While both primary and recurrent tumors contained cholesterol, CEs were detected exclusively in the recurrent tumor (**Figure 3A**). The CE/cholesterol ratio in the recurrent tumor was 0.44, indicating that approximately 30% of the cholesterol pool was esterified. In an autopsied GBM specimen from an end-stage patient, this ratio further increased to ~70%, underscoring progressive accumulation of CEs during disease advancement.

Cholesterol esterification is mediated by the enzyme acyl-CoA:cholesterol acyltransferase (ACAT) in the endoplasmic reticulum and serves as a mechanism for storing excess cholesterol in lipid droplets to support membrane synthesis in proliferating cells (**Figure 3C**). Although tightly regulated under physiological conditions, ACAT activity is dysregulated in cancer, including GBM, where ACAT1 is frequently overexpressed and CEs get accumulated (21–24). Lipid droplet formation and CE accumulation have been proposed as hallmarks of GBM, and several studies have demonstrated that ACAT inhibition suppresses CE synthesis and reduces tumor cell viability in GBM models (25–28). Our findings further support the involvement of cholesterol esterification in glioma progression and suggest that this pathway may be targetable (29).

In conclusion, our study reveals significant metabolic alterations associated with glioma progression from diffuse astrocytoma with TERTp mutation (molecular GBM) to WHO Grade 4 GBM. In particular, elevated levels of alanine and accumulation of cholesteryl esters were prominent features of the recurrent tumor. These findings suggest that alanine may serve as both a metabolic driver and biomarker of aggressive tumor behavior, while CE accumulation highlights cholesterol esterification as a metabolic vulnerability in GBM.

We are currently expanding our study to include a larger cohort of glioma patients, encompassing various molecular subtypes (e.g., IDH-mutant astrocytomas and oligodendrogliomas), and are generating patient-derived glioma cell lines for *in vitro* assessment of metabolic pathways and therapeutic response to ACAT inhibitors. Follow-up *in vivo* studies using patient-derived xenograft (PDX) models are also planned to validate cholesterol esterification as a therapeutic target.

Ultimately, integrating metabolic profiling with molecular and histopathological analyses may improve diagnostic accuracy and guide the development of novel metabolism-targeted therapies for gliomas—including GBMs and recurrent molecular GBMs.

## Methods

### Sampling of tumor tissue specimens

Tumor tissue specimens were collected during the initial surgical resection in April 2018 from four distinct spatial locations, and from one additional location during a second surgery in February 2022. All specimens were obtained under image guidance using the Medtronic AxiEM™ workstation and the StealthStation^®^ navigation system (30). The sampling sites are illustrated as insets in **Figure 2B**.

### Chemicals and reagents

Methanol, chloroform, and deuterated chloroform (CDCl_3_) were obtained from Millipore Sigma (St. Louis, MO, USA). CDCl_3_ containing 0.05% tetramethylsilane (TMS), as well as deuterium oxide (D_2_O), deuterium chloride (DCl), and sodium deuteroxide (NaOD), were purchased from Cambridge Isotope Laboratories (Tewksbury, MA, USA). A 3-(trimethylsilyl)-1-propane sulfonic acid-d_6_ sodium salt (DSS-d_6_) solution was acquired from Chenomx Inc. (Edmonton, Canada).

### Methanol–chloroform-water extraction of tumor tissues

Approximately 50 mg of each tissue specimen was subjected to metabolite extraction using a methanol-chloroform-water extraction protocol, as previously described (31). The resulting aqueous methanol and chloroform phases were separated and were dried using a vacuum concentrator (CentriVap^®^, Labconco, Kansas City, MO). The residues from the aqueous-methanol phase were reconstituted in 180 µL of D_2_O containing 1.0 mM DSS-d_6_ (internal standard), and the pH was adjusted to 7.4. The residues from the chloroform phase were dissolved in 180 µL of CDCl_3_ containing 0.05% TMS (internal standard). All prepared samples were transferred to 3.0 mm NMR tubes for ^1^H NMR data acquisition.

### ^1^H NMR experiments

One-dimensional (^1^H) NMR spectra were acquired using a Bruker 800 MHz spectrometer equipped with a cryogenically cooled probe optimized for ^1^H/^13^C detection (Bruker Biospin, Billerica, MA). Data were collected using a nuclear Overhauser effect spectroscopy (NOESY) pulse sequence with water suppression. Acquisition parameters were as follows: number of scans = 128; 90° pulse width = 8.1 µs; number of time-domain points = 64k; inter-pulse delay = 5.0 s; spectral width = 9615 Hz; acquisition time = 3.4 s; and mixing time = 100 ms. Prior to Fourier transformation, the free induction decay (FID) data were multiplied by an exponential window function with a 0.3 Hz line broadening. Spectral processing and analysis were performed following protocols established in our prior studies (31, 32).

## Data Availability

The original contributions presented in the study are included in the article/**Supplementary Material**. Further inquiries can be directed to the corresponding authors.
