# Supplementary material for: Case Report: Metabolic alterations and cholesterol esterification in a low-grade diffuse astrocytoma patient who progressed to glioblastoma at recurrence
Source: Front Oncol. 2025 Sep 19;15:1651974. doi: 10.3389/fonc.2025.1651974 (PMC12491059; doi:10.3389/fonc.2025.1651974)
Supplement: Supplementary file 1 [file DataSheet1.docx]

**Case Report: Metabolic alterations and cholesterol esterification in a low-grade diffuse astrocytoma patient who progressed to glioblastoma at recurrence**

Omkar B. Ijare^1,2,3,4,5^*, David S. Baskin^1,2,3,4,5,6^ , Suzanne Z. Powell^2,3,4,7,5^, and Kumar Pichumani^1,2,3,4,5^*

^1^Kenneth R. Peak Brain and Pituitary Tumor Treatment Center, Department of Neurosurgery,

Houston Methodist Hospital, Houston, TX, United States,

^2^Houston Methodist Academic Institute, Houston, TX, United States,

^3^Houston Methodist Research Institute, Houston, TX, United States,

^4^Houston Methodist Neurological Institute, Houston, TX, United States,

^5^Weill Cornell Medical College, New York, NY United States, and

^6^Texas A&M University College of Medicine, Houston, TX, United States,

^7^Department of Pathology and Genomic Medicine, Houston Methodist Hospital, Houston, TX, United States

***Correspondence to:**

Kumar Pichumani, Ph.D., Tel: 713 441 7190; E-Mail: [kpichumani@houstonmethodist.org](mailto:kpichumani@houstonmethodist.org)

and Omkar B. Ijare, Ph.D., Tel: 713 441 3547; E-Mail: [oijare@houstonmethodist.org](mailto:oijare@houstonmethodist.org)

***In vivo* ^1^H magnetic resonance spectroscopy (MRS) and CT/PET**


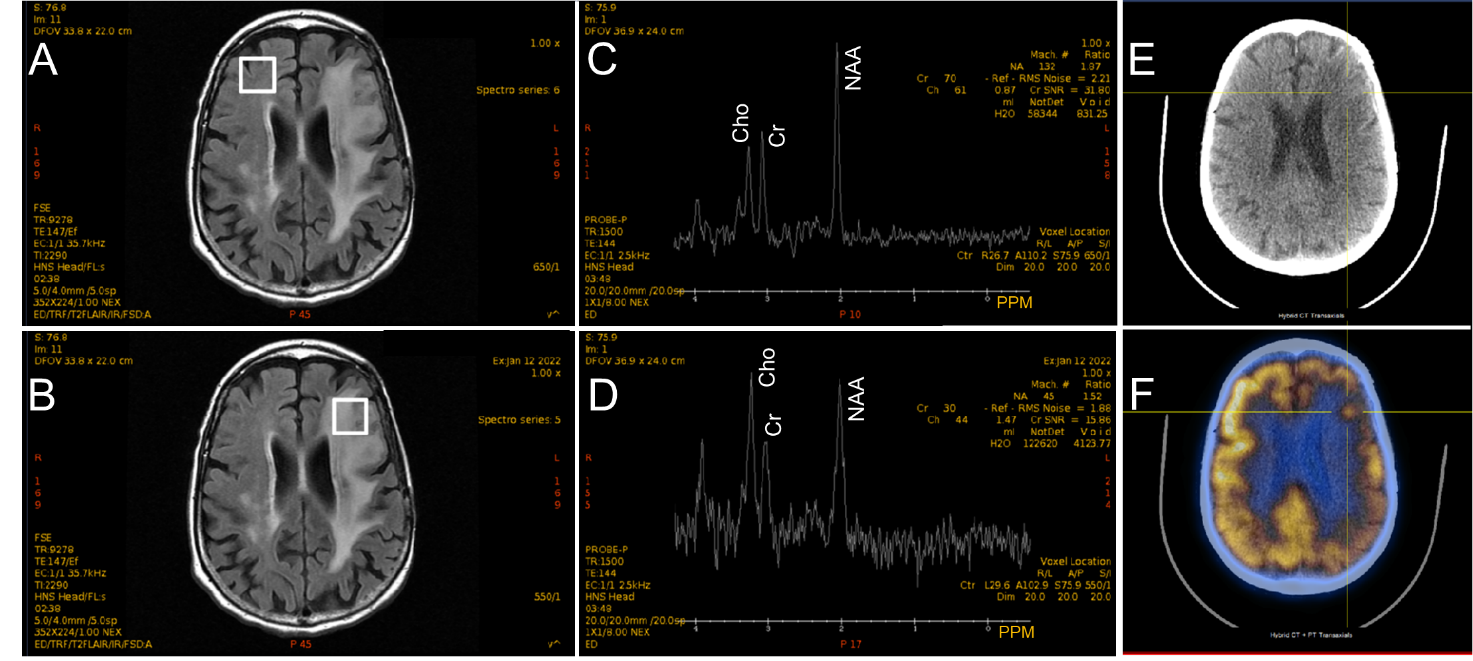


**Figure S1:** *In vivo* ^1^H MRS and ^18^F-FDG CT/PET data from the patient showing metabolic profiles of the tumor and non-tumor regions of the brain including ^18^F-FDG uptake by the tumor. (A) and (B) show axial slices of T2-FLAIR MR images and the location of the spectroscopic voxels (voxel size: 20x20x20 mm^3^) in the non-tumor and tumor regions of the patient brain. (C) and (D) show the *in vivo* ^1^H MR spectral profiles of non-tumor and tumor regions (TE = 144 ms; TR = 1500 ms). Figure 2D clearly shows the elevated levels of choline (Cho) and decreased levels of NAA, which are characteristics of a high-grade malignant glioma. (E) and (F) depict CT and CT/PET images showing the uptake of ^18^F-FDG by the recurrent tumor (crosshairs in Figure 2F).

The *in vivo* ^1^H magnetic resonance spectroscopy (MRS) of the recurrent tumor showed decreased levels of N-acetylaspartate (NAA) and elevated levels of choline, consistent with the high-grade glioma (Figure S1: D). The Choline-to-NAA (Cho/NAA) ratio which is considered to be a measure of tumor proliferation index was calculated from the peak intensities of the respective metabolite peaks from the non-tumor and tumor regions of the brain (Figure S1: C & D). This ratio for the tumor region was found to be 0.98, while for the non-tumor region this ratio was 0.46. Moreover, the ^18^F-fluorodeoxy glucose (^18^F-FDG) positron emission tomography (PET) performed when the patient showed tumor recurrence also showed abnormal uptake of ^18^F-FDG in the inferior left frontal lobe (Figure S1: F) close to the contrast enhanced tumor region in T1w MRI (Figure S1: E), suggesting the presence of malignant tissue in this region.
